# Optical Coherence Tomography (OCT) Findings in Post-COVID-19 Healthcare Workers

**DOI:** 10.3390/jimaging11060195

**Published:** 2025-06-12

**Authors:** Sanela Sanja Burgić, Mirko Resan, Milka Mavija, Saša Smoljanović Skočić, Sanja Grgić, Daliborka Tadić, Bojan Pajic

**Affiliations:** 1 Faculty of Medicine, University of Banja Luka, 78000 Banja Luka, Bosnia and Herzegovina; 2 Eye Clinic, University Clinical Center of Republic of Srpska, 78000 Banja Luka, Bosnia and Herzegovina; 3 Medical Faculty of the Military Medical Academy, University of Defence in Belgrade, 11000 Belgrade, Serbia; 4 Eye Clinic, Military Medical Academy in Belgrade, 11000 Belgrade, Serbia; 5 Department of Physics, Faculty of Scienses, University of Novi Sad, 21000 Novi Sad, Serbia; 6 Faculty of Medicine, University of Novi Sad, 21000 Novi Sad, Serbia; 7 Eye Clinic, King Khalid Hail Hospital, Hail 55421, Saudi Arabia; 8 Neurology Clinic, University Clinical Center of Republic of Srpska, 78000 Banja Luka, Bosnia and Herzegovina; 9 Division of Ophthalmology, Department of Clinical Neurosciences, Geneva University Hospitals, 1211 Geneva, Switzerland; 10 Experimental Ophthalmology, University of Geneva, 1211 Geneva, Switzerland; 11 Eye Clinic ORASIS, Swiss Eye Research Foundation, 4153 Reinach, Switzerland

**Keywords:** optical coherence tomography, post-COVID-19 retinal changes, neurovascular imaging

## Abstract

Recent evidence suggests that SARS-CoV-2 may induce subtle anatomical changes in the retina, detectable through advanced imaging techniques. This retrospective case–control study utilized optical coherence tomography (OCT) to assess medium-term retinal alterations in 55 healthcare workers, including 25 individuals with PCR-confirmed COVID-19 and 30 non-COVID-19 controls, all of whom had worked in COVID-19 clinical settings. Comprehensive ophthalmological examinations, including OCT imaging, were conducted six months after infection. The analysis considered demographic variables, comorbidities, COVID-19 severity, risk factors, and treatments received. Central macular thickness (CMT) was significantly increased in the post-COVID-19 group (*p* < 0.05), with a weak but statistically significant positive correlation between CMT and disease severity (r = 0.245, *p* < 0.05), suggesting potential post-inflammatory retinal responses. No significant differences were observed in retinal nerve fiber layer (RNFL) or ganglion cell complex (GCL + IPL) thickness. However, mild negative trends in inferior RNFL and average GCL+IPL thickness may indicate early neurodegenerative changes. Notably, patients with comorbidities exhibited a significant reduction in superior and inferior RNFL thickness, pointing to possible long-term neurovascular impairment. These findings underscore the value of OCT imaging in identifying subclinical retinal alterations following COVID-19 and highlight the need for continued surveillance in recovered patients, particularly those with pre-existing systemic conditions.

## 1. Introduction

Severe acute respiratory syndrome coronavirus 2 (SARS-CoV-2) has affected millions globally, with healthcare workers (HCWs) being among the most vulnerable groups due to high exposure. A 2025 global meta-analysis reported that SARS-CoV-2 infection rates among HCWs reached 11%, underscoring the occupational risks they faced during the pandemic [1]. Although the respiratory and systemic effects of COVID-19 are well-documented, growing evidence suggests ophthalmic implications, particularly for the retinal microvasculature. Angiotensin-converting enzyme 2 (ACE2), a key receptor for SARS-CoV-2 [2], is part of the renin–angiotensin system (RAS), which regulates blood pressure and fluid balance. RAS also plays a proinflammatory role in ocular disorders like diabetic and hypertensive retinopathy. ACE2 is widely expressed in ocular tissues, including the cornea, conjunctiva, aqueous humor, and retina, with studies detecting SARS-CoV-2 nucleic acid in retinal tissue [3,4,5,6]. Multiple mechanisms may contribute to retinal involvement in COVID-19, including direct infection via the ocular surface, neuronal retrograde transmission through the optic nerve, and hematogenous spread via ACE2 and CD147 receptors on retinal endothelial cells [7,8,9]. These pathways can trigger inflammation, endothelial dysfunction, thrombosis, and ischemia, potentially leading to retinal vasculitis and microvascular damage [10].

Diabetes mellitus, hypertension, and respiratory diseases have been shown to be among the key risk factors for COVID-19. Several studies have investigated retinal involvement in COVID-19 patients [11,12,13,14]. This is particularly important for COVID-19 patients with pre-existing retinopathy. Diabetic patients with COVID-19 may have an increased risk of retinal involvement and worsening of existing diabetic retinopathy due to direct activation of the transmembrane glycoprotein CD147 expressed mainly in retinal ganglion cells or indirectly through the cytokine storm associated with COVID-19 [15].

Optical coherence tomography (OCT) is a non-invasive, non-contact imaging technique that provides real-time, high-resolution cross-sectional images of ocular tissues. Based on low-coherence interferometry with near-infrared light, it enables detailed in vivo visualization of retinal microstructures at depths of around 1–2 mm, with axial resolution ranging from 1 to 10 μm. Clinically, OCT is essential for the early diagnosis, monitoring, and treatment evaluation of retinal diseases.

Recent studies have begun to explore retinal involvement in COVID-19, but findings have varied depending on imaging modality, study population, and methodology. Savastano et al. first reported hyperreflective lesions in the ganglion cell layer and inner plexiform layer in post-COVID-19 patients, suggesting possible neuroinflammatory processes [16]. However, their sample size was limited, and no follow-up was performed. Marinho et al. described cotton wool spots and microhemorrhages using fundus photography in COVID-19 patients, though without OCT or angiographic evaluation, limiting the structural interpretation [17]. Lani-Louzada et al. employed OCT angiography (OCTA) and reported reduced vessel density in both superficial and deep capillary plexuses among COVID-19 patients. However, the absence of a control group restricted the generalizability of their findings [18]. Abrishami et al. similarly used OCTA and found significant macular perfusion deficits in recovered patients [19], but the study lacked corresponding structural OCT analysis, which would have enhanced interpretation. Mavi Yildiz et al. conducted a spectral-domain OCT study and observed changes in central macular thickness and RNFL [20], although variability in disease severity and the absence of detailed systemic profiling limited causal inferences. More recently, Kaim et al. demonstrated thinning of the ganglion cell layer in post-COVID-19 patients, particularly in those with severe disease [21]. While informative, the cohort included a broad range of cases and was not limited to healthcare workers. Lyons et al. examined non-hospitalized Long COVID-19 patients using OCTA and found decreased retinal capillary perfusion [22]. However, they did not correlate perfusion changes with structural OCT parameters. Taken together, existing studies suggest that COVID-19 may lead to both vascular and structural retinal changes. However, most prior studies lacked uniform clinical stratification, matched control groups, or occupationally defined cohorts.

OCT has been utilized as a non-invasive tool to detect subclinical retinal alterations in post-COVID-19 patients. This study aims to evaluate medium-term OCT findings in healthcare workers following SARS-CoV-2 infection, investigating associations with disease severity, comorbidities, treatment received, smoking status, and demographic factors. In contrast to previous studies, our work focuses on a well-defined occupational cohort (healthcare workers), includes a matched control group, and applies standardized OCT protocols with clinical stratification. This enables a more specific interpretation of potential post-COVID-19 retinal changes.

To our knowledge, this is one of the few studies that focuses exclusively on healthcare workers and uses OCT to assess post-COVID-19 retinal changes. The findings aim to contribute to the understanding of potential neurovascular involvement in a high-risk occupational group.

## 2. Materials and Methods

### 2.1. Study Design and Participants

This was a single-center retrospective comparative case–control study conducted in a tertiary healthcare facility, the University Clinical Center of Republic of Srpska, Bosnia and Herzegovina. The study cohort included 55 unvaccinated healthcare workers (physicians and nurses), all of whom had worked at COVID-19 outpatient and inpatient clinics at the University Clinical Center since the outbreak of COVID-19 from 4 March 2020 until the end of 2020. At the time of this study, vaccination was not available, meaning all participants were unvaccinated. Participants were divided into two groups:

- Post-COVID-19 group (*n* = 25): HCWs with a prior PCR-confirmed SARS-CoV-2 infection.
- Non-COVID-19 group (*n* = 30): HCWs without prior COVID-19 infection.

Inclusion criteria for the post-COVID-19 group were a positive PCR test for SARS-CoV-2, confirmed recovery, and a minimum of six months post-infection. The control group consisted of HCWs who had never tested positive for COVID-19. Exclusion criteria for both groups included pre-existing ocular diseases, systemic inflammatory disorders, retinal pathologies unrelated to COVID-19, any previous retinal treatment (laser photocoagulation, vitreoretinal surgery, intravitreal therapy), any intraocular surgery, prior diagnosis of glaucoma, ocular hypertension, uveitis, refractive error > ±6D, and OCT signal strength < 7.

Both eyes were included in the analysis. A posthoc comparison revealed no significant differences between right and left eyes in OCT parameters, and each eye was treated as an independent data point.

### 2.2. Ophthalmological Examination and OCT Analysis

All participants underwent clinical history (COVID-19 illness symptoms and severity, disease course and duration, relevant past medical history and comorbidities, smoking history) and comprehensive ophthalmological examination, including best-corrected visual acuity (BCVA), intraocular pressure (IOP) measurement, slit-lamp biomicroscopy, and dilated fundus examination. Optical coherence tomography scans were performed using spectral-domain OCT (SD-OCT—Zeiss Cirrus HD-OCT, Carl Zeiss Meditec AG, Germany) to evaluate retinal nerve fiber layer (RNFL) thickness, ganglion cell complex (GCL+IPL) thickness, and central macular thickness(CMT) changes at six months post-COVID-19 recovery.

The Zeiss Cirrus HD-OCT is an advanced spectral-domain OCT device used for high-resolution imaging of the macula and optic nerve head. It delivers detailed cross-sectional views with an axial resolution of around 5 microns and can perform up to 27,000 A-scans per second. The system includes various scanning protocols enabling a thorough evaluation of both macular and optic nerve areas.

The six-month follow-up period was chosen to allow for the resolution of acute inflammatory changes and to assess potential long-term structural alterations in the retina, as previous studies have suggested that post-viral neurovascular effects may take several months to manifest [23].

OCT Protocols:

- Macular thickness measurements were performed using the “Macular Cube 512 × 128 A-scan” protocol covering a 6 mm × 6 mm scanning area, divided into 9 zones according to the Early Treatment Diabetic Retinopathy Study (ETDRS) grid. All values, including CMT and parafoveolar and perifoveolar thickness, were automatically calculated by the Zeiss Cirrus HD-OCT system using built-in analysis software (11.5.2.54532).
- Ganglion Cell Complex Analysis: Based on a macular protocol centered on the fovea with automated (GCL+IPL) measurements divided into 6 quadrants.
- Optic Nerve Head and RNFL Thickness: Optic Disc Cube 200 × 200 protocol measuring RNFL thickness for a 2.4 mm diameter circle around the optic disc.

**Figure 1:**
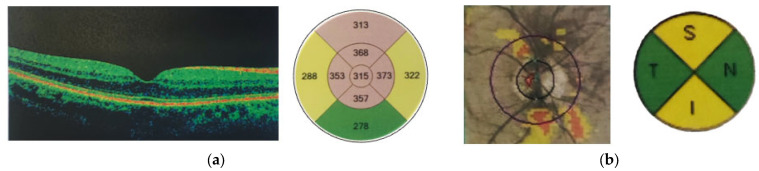
Spectral-domainoptical coherence tomography(SD-OCT)of the macula (**a**) and optic nerve head (**b**) of a post-COVID-19 participant; a Zeiss Cirrus HD-OCT machine (Carl Zeiss Meditec AG, Germany) was used.

Representative spectral-domain optical coherence tomographzy images of the macula and optic nerve head obtained using the Zeiss Cirrus HD-OCT system are shown in Figure 1, illustrating the imaging protocol and quality used in this study.

Demographic and clinical data, including COVID-19 severity and treatment, were collected from medical records. COVID-19 diagnosis and classification were based on WHO guidance [24].

The standard treatment in this study referred to supportive care measures, including symptomatic treatment (e.g., antipyretics, analgesics, and hydration), as well as, in some cases, empiric antibiotic therapy without the use of antiviral or immunomodulatory agents such as hydroxychloroquine, tocilizumab, or corticosteroids.

This study was conducted in accordance with the Declaration of Helsinki and approved by Ethics Committee of the University Clinical Center of Republic of Srpska (approval number 01-19-116-2/25).

### 2.3. Dataset Description

The dataset consisted of 55 participants, including 25 post-COVID-19 healthcare workers and 30 non-COVID-19 controls. All participants underwent comprehensive ophthalmic examinations using SD-OCT. The dataset contained demographic data (age, sex), clinical parameters (presence of comorbidities, smoking status), disease-related information (COVID-19 severity, symptoms, treatment), and OCT-derived retinal measurements. OCT parameters analyzed included central macular thickness (CMT); parafoveolar and perifoveolar retinal thickness in the superior, temporal, inferior, and nasal sectors; cube volume and cube average thickness; retinal nerve fiber layer (RNFL) thickness in the superior, temporal, inferior, and nasal quadrants; and ganglion cell complex (GCL+IPL) thickness (average and minimum).

In addition to structural parameters, the dataset contained treatment information (e.g., hydroxychloroquine, azithromycin, corticosteroids, immunotherapy) and self-reported symptom data. COVID-19 severity was stratified according to WHO clinical criteria into asymptomatic, mild, moderate, severe, and critical categories.

The dataset was cleaned and structured prior to analysis. Missing entries were excluded from statistical calculations. A preliminary data exploration was conducted using violin plots to visualize the distribution of key OCT features across study groups, as shown in Supplementary Figure S1.

### 2.4. Statistical Analysis

Data were analyzed using statistical software SPSS v23.0, with comparisons between post-COVID-19 and non-COVID-19 groups performed using *t*-tests for continuous variables and chi-square tests for categorical variables. The Shapiro–Wilk test was used to assess the data distribution. Parametric tests were used for a normal distribution. Data that did not follow a normal distribution were analyzed using the Mann–Whitney U test. ANOVA was used to analyze differences across groups. Multivariate regression analysis was conducted to determine the impact of gender, disease severity, and systemic risk factors on OCT parameters. Correlations (Pearson correlation) between the COVID-19 severity score and OCT findings were also analyzed. A significance level of *p* <0.05 was considered statistically significant.

### 2.5. Software and Visualization Tools

Supplementary visualizations, including violin plots used for exploratory data analysis, were created in Python 3.10 using the pandas, seaborn, and matplotlib libraries. These plots were used solely for data visualization.

## 3. Results

### 3.1. Demographic and Clinical Characteristics

A total of 110 eyes of 55 Caucasian subjects were included in this study. The mean age of the post-COVID-19 group was 37.68 ± 10.12 years, and the non-COVID-19 group was 38.92 ± 11.06 years. In the post-COVID-19 group, 72% were males and 28% were females. COVID-19 symptoms were exhibited by 84% of post-COVID-19 patients, with the most common being fever, while 56% had mild COVID-19 disease based on WHO clinical criteria [16].Comorbidities such as cardiovascular diseases and endocrine diseases were recorded in both groups. Further details can be found in Table 1.

### 3.2. OCT Findings

OCT findings indicated that CMT was significantly higher in post-COVID-19 patients (*p* < 0.05).However, no statistically significant differences were found in RNFL and GCL + IPL thickness between groups. Further details can be found in Table 2.

Table 2 presents aquantitative analysis of OCT parameters, comparing thepost-COVID-19 and non-COVID-19 groups. Across all measured quadrants (superior, temporal, inferior, and nasal), no statistically significant differences were observed between groups (*p* > 0.05). However, a slight trend of decreased inferior RNFL thickness was noted in post-COVID-19 patients. The average and minimum GCL+IPL thickness did not significantly differ between groups (*p* > 0.05). While cube volume appeared slightly higher in the post-COVID-19 group, cube average thickness showed no notable differences.

Multivariate regression analysis showed that gender had no statistically significant effect on any of the analyzed OCT parameters (*p* > 0.05).

There was no statistically significant difference in subgroup analyses based on age. Although a trend of increased CMT was observed in younger post-COVID-19 patients, the difference did not reach statistical significance (*p* > 0.05). Similarly, no significant differences were observed in RNFL or GCL+IPL thickness between younger and older subgroups (Table 3).

Patients without comorbidities did not show significant OCT differences between groups. However, in patients with comorbidities, post-COVID-19 patients exhibited increased CMT (*p* < 0.01) and parafoveolar temporal thickness (*p* < 0.05), along with a decrease in superior and inferior RNFL thickness (*p* < 0.05, *p* < 0.001) (Table 4).

Treatment type also influenced inferior RNFL thickness. Patients who received hydroxychloroquine and tocilizumab had significantly lower inferior RNFL thickness compared to those who received no or standard treatment (*p* < 0.05, ANOVA) (Table 5).

**Figure 2:**
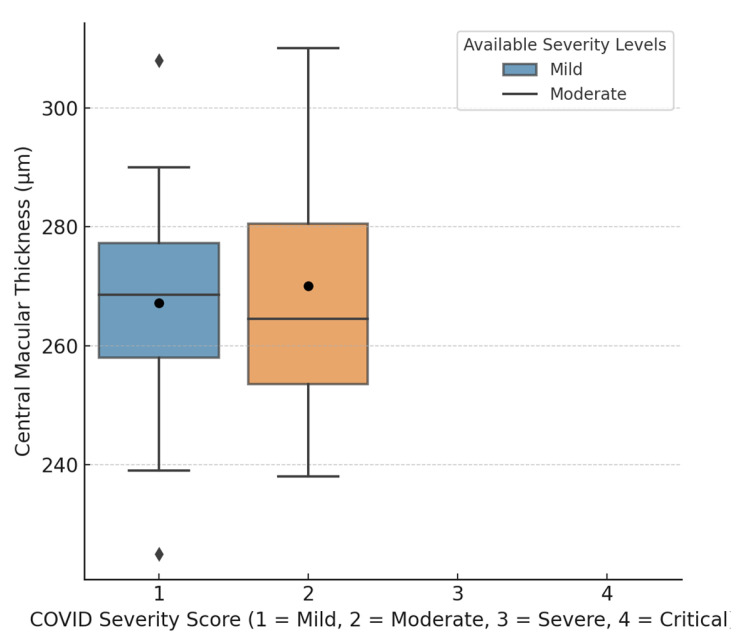
Box plot showing the distribution of central macular thickness (CMT) across COVID-19 severity levels. Although group-wise differences were not statistically significant, a separate correlation analysis revealed a statistically significant positive relationship between CMT and COVID-19 severity (r = 0.245, *p* = 0.02), suggesting that increased COVID-19 severity is associated with greater CMT.

**Figure 3:**
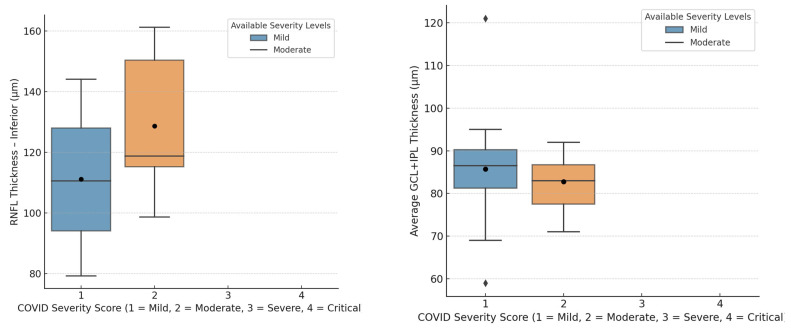
(**a**) Box plot illustrating the distribution of inferior retinal nerve fiber layer (RNFL) thickness across COVID-19 severity levels. The median values appear slightly higher in the moderate group, but the interquartile overlap and broad data spread suggest no statistically significant difference across groups. A separate correlation analysis showed a weak negative association between inferior RNFL thickness and the COVID-19 severity score (r = −0.151), indicating a subtle trend toward thinning with increased disease severity.(**b**) Box plot of the distribution of the average ganglion cell layer + inner plexiform layer (GCL+IPL) thickness across COVID-19 severity levels. Median and mean values are marginally lower in the moderate group, but with overlapping distributions. Correlation analysis also revealed a slight negative trend between the average GCL+IPL thickness and the COVID-19 severity score (r = −0.176), though this trend was not statistically significant.

A positive correlation (r = 0.245, *p* =0.02) was found between COVID-19 severity and CMT (Figure 2). Slight negative trends in inferior RNFL (−0.151) and average GCL + IPL thickness (−0.176) were observed, though these were not statistically significant (Figure 3).

In the post-COVID-19 group, smoking significantly affected parafoveolar superior and parafoveolar temporal macular thickness (*p* < 0.05), indicating a potential risk factor for retinal structural alterations, while in the non-COVID-19 group, nasal RNFL thickness was significantly affected (*p* < 0.05). However, no other OCT parameters show significant differences between smokers and non-smokers in the non-COVID-19 group (Table 6).

## 4. Discussion

Healthcare workers were at increased risk of SARS-CoV-2 exposure during the pandemic [25,26,27], making them an important cohort for studying post-COVID-19 effects, including retinal changes. SARS-CoV-2 can be transmitted by symptomatic patients; however, asymptomatic infected patients have similar transmission potential due to viral loads similar to those detected in symptomatic patients [28]. Several reports have highlighted ophthalmic manifestations of COVID-19, primarily conjunctivitis, vascular occlusions, and neuroinflammatory changes [11,12,13,14,17,29,30,31,32,33,34]. However, studies investigating subclinical OCT changes in post-COVID-19 patients remain limited.

Our study found a significant increase in CMT in post-COVID-19 patients compared to non-COVID-19 controls (*p* < 0.05). This increase in macular thickness may suggest post-inflammatory retinal changes due to SARS-CoV-2 infection. This aligns with findings from previous research, where CMT increases were attributed to inflammatory and microvascular alterations post-infection [20]. The mechanism behind this thickening may involve endothelial dysfunction, immune-mediated inflammation, or retinal ischemia due to SARS-CoV-2-related ACE2 downregulation [35]. While cube volume appeared slightly higher in the post-COVID-19 group, cube average thickness showed no notable differences, further supporting the hypothesis that macular changes are localized rather than widespread across the retina. However, another longitudinal study indicated significant thinning in the temporal and superior quadrants of the macula and RNFL regions six months post-infection, further supporting the hypothesis that COVID-19 may lead to progressive retinal change over time [36].

In contrast, we found no significant differences in RNFL or GCL+IPL thickness between groups, indicating that widespread neurodegeneration may not be a major feature in this cohort. These results are consistent with studies where no RNFL or GCL+IPL thinning was observed in mild COVID-19 cases [20,37]. However, some studies reported RNFL thinning in patients with neurological COVID-19 symptoms, likely due to neurovascular alterations [38,39,40]. Detailed segmentation analysis showed thinning of the GCL+IPL and INL (inner nuclear layer) in COVID-19 patients with neurological symptoms [41]. Our findings suggest that neurodegenerative changes may be more pronounced in severe COVID-19 cases, a hypothesis that warrants further investigation. A slight trend of decreased inferior RNFL thickness was notedin our studyin post-COVID-19 patients, which could indicate potential subclinical neurodegenerative effects. The average and minimum GCL+IPL thickness did not significantly differ between groups (*p* > 0.05). This suggests that ganglion cell layer and inner plexiform layer integrity was largely preserved in post-COVID-19 individuals.

Our results indicated a positive association between the severity of COVID-19 and increased CMT, which may reflect underlying post-inflammatory retinal alterations. Additionally, a mild decreasing trend in inferior RNFL and average GCL+IPL thickness was noted, potentially pointing to subtle neurovascular changes, although these findings did not reach statistical significance.

Comorbidities played a crucial role in retinal changes. Post-COVID-19 patients with pre-existing comorbidities exhibited significant increases in CMT and decreases in superior and inferior RNFL thickness. These findings align with prior reports linking systemic inflammation, vascular damage, and retinal alterations in patients with systemic vascular and neurovascular diseases [42]. However, CMT thinning, typically linked to cardiovascular risk factors, was not consistent with our results [43].

Our findings align with several recent studies investigating structural retinal changes in post-COVID-19 patients. Kaim et al. [21] reported significant thinning of the RNFL and GCL, particularly in patients with severe symptoms, which is consistent with our observation of superior and inferior RNFL thinning in patients with comorbidities. Similarly, Talebnejad et al. [44] demonstrated sublayer-specific retinal changes associated with COVID-19 severity, further supporting the idea of a neuroretinal impact.

Regarding central macular thickness, our data showed a significant increase in CMT among post-COVID-19 participants, echoing findings by Mavi Yildiz et al. [20], who observed comparable macular thickening in recovered patients. In contrast, we found no significant differences in average GCL + IPL thickness, which differs from the global thinning reported in some earlier studies [21,22], potentially due to differences in population characteristics or OCT segmentation protocols.

Studies have shown that smokers exhibit increased ACE2 expression, which may heighten their risk of SARS-CoV-2 infection [45]. It was observed that healthy chronic smokers had reduced RNFL thickness while CMT remained unchanged, indicating that RNFL alterations may be linked to endothelial dysfunction and changes in retinal vascular reactivity due to smoking [46]. In our study, smoking was found to significantly affect central superior and central temporal macular thickness (*p* < 0.05) in the post-COVID-19 group, suggesting it may be a contributing risk factor for retinal structural alterations in these patients. In contrast, within the non-COVID-19 group, only nasal RNFL thickness showed a significant difference between smokers and non-smokers (*p* < 0.05). No other OCT parameters demonstrated statistically significant differences in the non-COVID-19 group, indicating a potentially stronger retinal impact of smoking in individuals with a history of COVID-19.

Interestingly, we observed that treatment with hydroxychloroquine and tocilizumab was associated with lower inferior RNFL thickness. Hydroxychloroquineis known for its potential retinal toxicity, although its long-term effects on post-COVID-19 patients remain unclear [47,48]. We found no reports of changes associated with tocilizumab [49].

It remains unclear whether the retinal changes observed at 6 months represent stable findings, progressive neurodegeneration, or reversible inflammatory responses. Future longitudinal studies are needed to determine the trajectory and permanence of these alterations, especially in individuals with systemic comorbidities or those treated with potentially retinotoxic therapies.

While previous studies reported decreased retinal vessel density on OCT angiography (OCTA) in post-COVID-19 patients [19,38,50], our study did not include OCTA, limiting our ability to assess microvascular perfusion changes. However, Lyons et al. [22] highlighted vascular perfusion deficits in non-hospitalized Long COVID-19 patients, suggesting that microvascular changes may precede or accompany structural alterations.

Future research should incorporate multimodal imaging to better understand SARS-CoV-2’s impact on retinal vascularization. In particular, OCTA enables non-invasive visualization and quantification of microvascular perfusion parameters, such as capillary density, flow voids, and foveal avascular zone dimensions, which may reveal subclinical ischemic or inflammatory changes before structural alterations become detectable on OCT. The integration of OCTA in future longitudinal assessments could therefore enhance our understanding of both early and progressive retinal vascular involvement in post-COVID-19 patients, especially those with systemic vascular comorbidities.

## 5. Conclusions

Our study demonstrates that post-COVID-19 patients, particularly those with pre-existing comorbidities, exhibit significant increases in central macular thickness compared to controls. Additionally, significant thinning of the superior and inferior RNFL was observed in post-COVID-19 patients with comorbidities, suggesting a possible neurovascular impact of the infection. However, no significant differences were found in average RNFL or GCL+IPL thickness, indicating that widespread retinal neurodegeneration is not a major feature in this cohort. Treatment-related changes in RNFL thickness highlight the need for longitudinal studies assessing the long-term impact of COVID-19 and its therapies on retinal structure. OCT has proven to be a valuable tool for detecting subtle changes in the posterior segment, providing critical insights into subclinical retinal alterations that could remain undetected without detailed imaging.

The limitations of our study include the retrospective study design, relatively small sample size, lack of longitudinal observation, and lack of a multimodal imaging approach. Furthermore, we do not possess OCT results obtained prior to the onset of disease. Therefore, we were not able to measure OCT changes before and after COVID-19 infections. To better determine whether the observed retinal changes are directly attributable to SARS-CoV-2 infection or influenced by other systemic factors, future studies should adopt a prospective design including baseline (pre-infection) OCT measurements. Further comprehensive analysis should be undertaken, and larger prospective studies will be required on SARS-CoV-2’s retinal impact. In addition, future studies may benefit from the integration of automated image analysis techniques, including segmentation, feature extraction, or deep-learning-based classification, which could enable the detection of subtle retinal changes beyond conventional measurement tools.

## Figures and Tables

**Table 1:** Demographic and clinical characteristics of post-COVID-19 and non-COVID-19 patients.

Characteristics	Post-COVID-19	Non-COVID-19
Sex		
Female	28%	88%
Male	72%	12%
Age (years)	37.68 ± 10.12	38.92 ± 11.06
Smoking		
Yes	24%	36%
No	76%	64%
Comorbidity		
Cardiovascular diseases	8%	12%
Endocrine diseases	16%	32%
Respiratory system diseases	8%	4%
Other	0%	4%
Clinical presentation		
Fever	76%	
Malaise	32%	
Anosmia	24%	
Ageusia	20%	
Severity of COVID-19		
Asymptomatic	16%	
Mild	56%	
Moderate	24%	
Severe	4%	
Critical	0%	
Treatment recieved		
No medication	36%	
Symptomatic	52%	
Hydroxychloroquine	20%	
Azytromycine	48%	
Remdesevir	0%	
Favipiravir	0%	
Methylprednisolone	8%	
Tocilizumab	12%	

**Table 2:** OCT findings in both groups, integrating the statistical analyses.

OCT Findings	Post-COVID-19	Non-COVID-19	*p*-Value
Central macular thickness (CMT)	272.94 ± 20.79 µm	259.99 ± 24.83 µm	<0.05 *
RNFL thickness			
Superior quadrant	115.02 ± 28.72 µm	120.95 ± 15.17 µm	>0.05
Temporal quadrant	62.08 ± 20.17 µm	67.03 ± 21.16 µm	>0.05
Inferior quadrant	120.94 ± 35.43 µm	130.00 ± 36.31 µm	>0.05
Nasal quadrant	72.90 ± 12.40 µm	71.98 ± 12.88 µm	>0.05
GCL+IPL thickness			
Average	85.00 ± 10.99 µm	84.81 ± 10.12 µm	>0.05
Minimum	80.96 ± 10.45 µm	81.38 ± 12.81 µm	>0.05
Cube volume	13.96 ± 3.75 mm^3^	10.43 ± 3.64 mm^3^	>0.05
Cube average thickness	287.04 ± 17.96 µm	288.98 ± 15.34 µm	>0.05

Asteriks (*) indicates statistical significance at *p* < 0.05. Bold values highlight statistically significant results.

**Table 3:** Comparison of CMT, RNFL, and GCL + IPL by age group.

	<38 Years Old	≥38 Years Old	*p*-Value
Central macular thickness (CMT)	269.44 ± 22.41 µm	265.95 ± 14.46 µm	>0.05
RNFL thickness			
Superior quadrant	118.82 ± 20.71 µm	109.00 ± 35.34 µm	>0.05
Temporal quadrant	59.46 ± 12.82 µm	63.10 ± 2.85 µm	>0.05
Inferior quadrant	119.34 ± 37.61µm	126.67 ± 15.50 µm	>0.05
Nasal quadrant	74.34 ± 10.12 µm	73.72 ± 13.52 µm	>0.05
Average GCL+IPL thickness	83.24 ± 10.04 µm	87.48 ± 12.24 µm	>0.05

**Table 4:** Comparison of post-COVID-19 vs. non-COVID-19 patients with comorbidities.

	Post-COVID-19 with Comorbidities	Non-COVID-19 with Comorbidities	*p*-Value
Central macular thickness (CMT)	282.12 ± 17.77 µm	264.09 ± 23.75 µm	<0.01 *
Parafoveolar superior sector	336.58 ± 21.35 µm	339.45 ± 23.48 µm	>0.05
Parafoveolar temporal sector	330.06 ± 6.93 µm	318.31 ± 29.02 µm	<0.05 *
Parafoveolar inferior sector	332.05 ± 21.12 µm	337.42 ± 24.61 µm	>0.05
Parafoveolar nasal sector	336.82 ± 24.55 µm	338.13 ± 26.24 µm	>0.05
RNFL thickness			
Superior quadrant	108.75 ± 25.75 µm	124.38 ± 19.46 µm	<0.05 *
Temporal quadrant	63.25 ± 14.16 µm	63.83 ± 11.14 µm	>0.05
Inferior quadrant	103.38 ± 8.40 µm	135.56 ± 9.75 µm	<0.001 *
Nasal quadrant	76.62 ± 14.85 µm	73.53 ± 5.22 µm	>0.05
GCL+IPL thickness			
Average	83.44 ± 13.57 µm	86.12 ± 10.59 µm	>0.05
Minimum	80.62 ± 10.12 µm	82.88 ± 9.49 µm	>0.05

* The thickness of the perifoveolar sectors was also analyzed, but no statistically significant differences were found.

**Table 5:** OCT parameters by treatment type.

	Hydroxychloroquine/Tocilizumab	No Treatment/Standard Treatment	*p*-Value
Central macular thickness (CMT)	271.06 ± 20.00 µm	267.85 ± 15.29 µm	>0.05
Parafoveolar superior sector	335.88 ± 11.35 µm	332.26 ± 17.28 µm	>0.05
Parafoveolar temporal sector	324.25 ± 10.26 µm	320.44 ± 12.17 µm	>0.05
Parafoveolar inferior sector	333.94 ± 18.20 µm	329.06 ± 17.63 µm	>0.05
Parafoveolar nasal sector	338.19 ± 10.82 µm	333.03 ± 24.08 µm	>0.05
RNFL thickness			
Superior quadrant	123.25 ± 14.43 µm	122.76 ± 41.68 µm	>0.05
Temporal quadrant	64.50 ± 3.85 µm	64.65 ± 16.23 µm	>0.05
Inferior quadrant	96.81 ± 7.65 µm	127.62 ± 35.56 µm	<0.05 *
Nasal quadrant	75.25 ± 12.07 µm	71.71 ± 7.87 µm	>0.05
GCL+IPL thickness			
Average	81.81 ± 7.45 µm	83.50 ± 10.20 µm	>0.05
Minimum	79.44 ± 7.27 µm	81.94 ± 10.19 µm	>0.05

* The thickness of the perifoveolar sectors was also analyzed, but no statistically significant differences were found.

**Table 6:** OCT parameters by smoking status in post-COVID-19 and non-COVID-19 patients.

OCT Parameters	Post-COVID-19		Non-COVID-19		*p*-Value	
	Smoker	Non-Smoker	Smoker	Non-Smoker	COVID	Non-COVID
Central macular thickness	263.80 ± 18.53 µm	268.09 ± 14.79 µm	237.00 ± 18.03 µm	269.43 ± 21.62 µm	>0.05	>0.05
Parafov. superior sector	327.40 ± 17.42 µm	334.90 ± 18.43 µm	309.02 ± 15.00 µm	332.50 ± 16.61 µm	<0.05 *	>0.05
Parafov. temporal sector	318.07 ± 14.05 µm	324.22 ± 12.62 µm	289.07 ± 15.01 µm	321.64 ± 15.75 µm	<0.05 *	>0.05
Parafov. inferior sector	323.55 ± 15.83 µm	330.01 ± 18.18 µm	303.01 ± 17.01 µm	330.14 ± 17.06 µm	>0.05	>0.05
Parafov. nasal sector	327.10 ± 20.89 µm	332.90 ± 23.20 µm	308.03 ± 19.08 µm	332.57 ± 18.22 µm	>0.05	>0.05
RNFL thickness						
Superior quadrant	113.88 ± 29.35 µm	115.69 ± 21.53 µm	117.09 ± 19.98 µm	114.50 ± 11.45 µm	>0.05	>0.05
Temporal quadrant	61.70 ± 11.44 µm	58.48 ± 12.46 µm	63.40 ± 9.89 µm	70.00 ± 8.15 µm	>0.05	>0.05
Inferior quadrant	108.90 ± 39.95 µm	104.28 ± 42.84 µm	138.11 ± 20.08 µm	129.86 ± 15.98 µm	>0.05	>0.05
Nasal quadrant	77.53 ± 12.41 µm	76.36 ± 10.66 µm	93.31 ± 13.92 µm	69.71 ± 6.59 µm	>0.05	<0.05 *
GCL + IPL thickness						
Average	84.97 ± 15.27 µm	82.65 ± 10.90 µm	78.21 ± 10.13 µm	84.50 ± 9.17 µm	>0.05	>0.05
Minimum	77.62 ± 11.20 µm	80.27 ± 13.30 µm	75.08 ± 11.02 µm	77.07 ± 19.34 µm	>0.05	>0.05

* The thickness of the perifoveolar sectors was also analyzed, but no statistically significant differences were found.

## Data Availability

All data generated or analyzed during this study are included in this published article. The datasets generated during and/or analyzed during the current study are available from the corresponding author on reasonable request.

## Supplementary Materials

The following supporting information can be downloaded at https://www.mdpi.com/article/10.3390/jimaging11060195/s1: Figure S1: Violin plots illustrating the distribution of selected OCT parameters in post-COVID-19 and non-COVID-19 participants. Parameters shown include central macular thickness (CMT), superior and inferior retinal nerve fiber layer (RNFL), and average ganglion cell complex (GCL+IPL) thickness. The violin plots depict the full distribution of values in each.
