# Supplementary material for: Optical Coherence Tomography (OCT) Findings in Post-COVID-19 Healthcare Workers
Source: J Imaging. 2025 Jun 12;11(6):195. doi: 10.3390/jimaging11060195 (PMC12193844; doi:10.3390/jimaging11060195)
Supplement: Supplementary file 1 [file jimaging-11-00195-s001.zip › jimaging-3624922-supplementary.pdf]

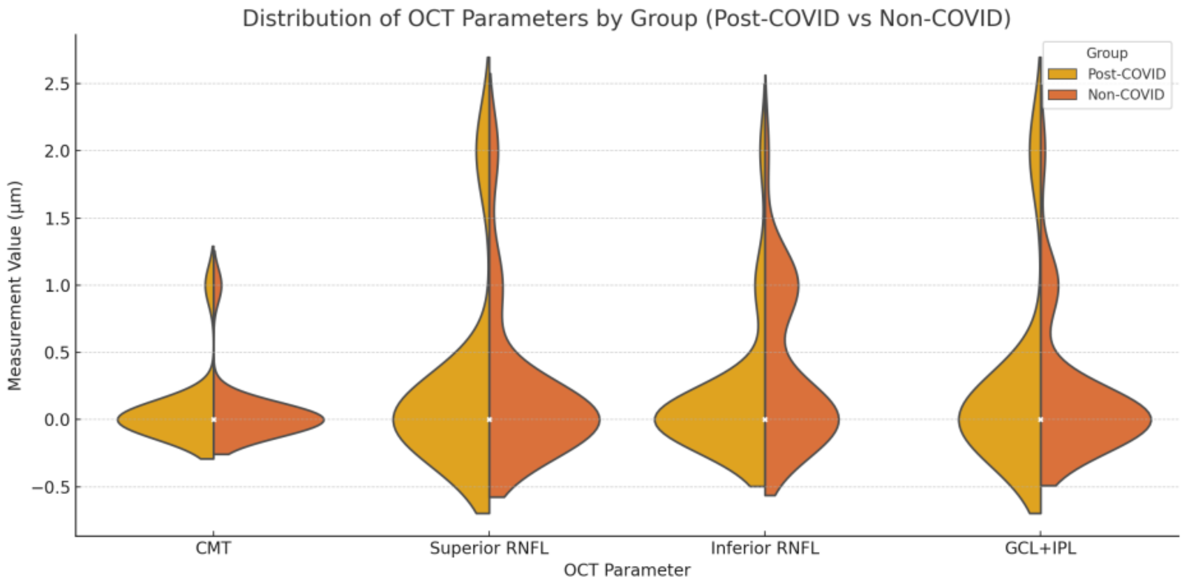

Supplementary Figure 1.

Violin plots illustrating the distribution of selected OCT parameters in post-COVID and non-COVID participants. Parameters shown include central macular thickness (CMT), superior and inferior retinal nerve fiber layer (RNFL), and average ganglion cell complex (GCL+IPL) thickness. The violin plots depict the full distribution of values in each.
